# Editorial: Insights in microbiological chemistry and geomicrobiology: 2022/2023

**DOI:** 10.3389/fmicb.2024.1446765

**Published:** 2024-07-17

**Authors:** Ruiyong Zhang

**Affiliations:** ^1^Key Laboratory of Advanced Marine Materials, Key Laboratory of Marine Environmental Corrosion and Bio-Fouling, Institute of Oceanology, Chinese Academy of Sciences, Qingdao, China; ^2^Guangxi Key Laboratory of Marine Environmental Science, Institute of Marine Corrosion Protection, Guangxi Academy of Sciences, Nanning, China

**Keywords:** extreme microbiology, microbial communities, biodegradation, geomicrobiology, biogeochemistry

Microbes are crucial in balancing and improving environmental systems in the lithosphere, hydrosphere, biosphere, and atmosphere, by shaping the surrounding geochemical and mineralogical components through their metabolic action (Druschel and Kappler, 2015). Further, the growth and metabolic activities of microbial communities are generally dictated by the surrounding environmental systems (Diener and Gibbons, 2023; Yang et al., 2023). Changes in the geochemical and mineralogical components of environmental systems, influenced by natural and anthropogenic activities, may affect the microbial communities. In particular, the resistance and adaptive ability of microbial communities to extreme conditions through diverse physiological and molecular functions have become crucial in mitigating and restoring ecosystems (Shade, 2023; Caro-Astorga et al., 2024; Song et al., 2024; Turrini et al., 2024). Exploring the interactions between microbial communities and geochemical components and other biota of different ecosystems, as well as uncovering the secrets hidden in them, is an ideal way to identify and solve problems in ecosystem management. This *Research Topic* “*Insights in microbiological chemistry and geomicrobiology: 2022/2023*” includes ten papers connected to the microbial community structure and assembly, physiological and molecular adaptive strategies to various extreme environments, and biogeochemical cycling role of the microbial community.

It is noteworthy that the composition and association of microbial communities are mainly affected by contaminants in ecosystems. Zhang W. et al. studied the diversity and composition of microbial communities in sediment and soil samples of heavily polluted estuarine karst environment and revealed that diversity, abundance, and structure of microbial communities are governed by the concentrations of organic pollutants [polycyclic aromatic hydrocarbons (PAHs) and hazardous trace elements (HTEs)] present in the estuarine environment. The variations in diversity and assembly of bacterial and fungal communities that respond to environmental factors at different altitudes and depths of mountain ecosystems were observed by Kang et al.. Their study revealed that the distribution of bacterial and fungal communities at soil depth varied and their assembly process was significantly related to DOC and C:N ratio of soil. For instance, bacterial community assembly processes were related to soil DOC and C:N ratio, while fungal community assembly was related to soil C:N ratio.

The diversity and abundance of microbes in ecosystems are influenced by environmental factors that vary over time and seasonally. Changes in environmental factors due to seasonally changing weather conditions can have negative effects on microbial communities. Xu et al. investigated the composition and distribution of *Massilia* bacterial species in constructed wetland ecosystems in different seasons and found that the richness of *Massilia* bacteria varied significantly in different environments and different seasons, with a greater abundance of *Massilia* bacterial species observed in summer and autumn than in spring and winter. Also, their study revealed that seasonal variation of bacterial species in different compartments of constructed wetland ecosystems correlated with environmental factors.

Various positive (mutualism, symbiosis, commensalism, and proto-cooperation) and negative (predation, parasitism, competition, and ammensalism) interactions between microbial communities and other biotas (animals, plants, humans, etc.) are driven by various physiological and molecular processes, which play an important role in balancing the ecosystems. Feng et al. investigated the relationships between epibiotic bacterial communities with various species of deep-sea squat lobsters (*Shinkaia crosnieri* and *Munidopsis Verrilli*) from cold-seep ecosystems and revealed the differences in epibiotic bacterial communities among the lobster species. Also, this study reported that epibiotic bacterial communities on lobster species use chemical fluxes near cold seeps in more efficient ways, which benefits the host's nutrient strategies.

Adaptation and resistance of microbial communities to extreme conditions through various internal and external processes are important in the environmental management and the bio-metallurgical field. In particular, the detoxification capability of indigenous microbial communities against organic and inorganic contaminants has attracted significant attention in the remediation of contaminated environments. Understanding the interactions and detoxification mechanisms of microbial communities on contaminants can help improve microbial bioremediation technology for environmental restoration. da Silva et al. studied the microbial action on reduction and oxidation of iron in hematite and goethite with four different treatment systems and found biofilm formation by microbes alters the levels of iron oxyhydroxides precipitation. Furthermore, their research found that iron-reducing families such as Enterobacteriaceae dominated the treatment samples. Their research results suggested that the ability of microbes to dissolve iron could help reduce the environmental effects of the iron mining industry and thereby restore the ecosystem. Shi et al. observed that glutathione reductase (GR) plays a key role in *Acidithiobacillus caldus* defense against reactive oxygen species caused by heavy metals.

Hydrothermal vent systems offer habitats that support microbial life due to their diverse chemical energy sources and steep physical and chemical gradients. Investigating the structure of microbial communities and their interaction with the physical and chemical gradients of hydrothermal vent systems helps to understand the biochemical cycling role of microbial communities in terrestrial and marine ecosystems. Nakano et al. used metagenomic analysis to identify magentosome-producing microorganisms, as well as mineralogical and geochemical studies to understand their role at deep-sea hydrothermal vent chimney samples from the South Mariana Trough. It is necessary to develop techniques to study microbial progress in the environment. Yin et al. developed a method of in situ and quantitative gas (methane) detection using Raman spectroscopy. This technology may be a powerful complementary tool to gas chromatography for monitoring microbiological progresses.

To verify whether the isotopic ratio in cell water is controlled by metabolic water produced by cellular respiration, Weiner et al. conducted experiments with varying the δ^18^O and by measuring the δ^18^O of cell phosphate. The authors provide evidence to show that the deviation from isotopic equilibrium between the ambient water and the oxygen in phosphate is not derived from the contribution of metabolic water to the cell water.

Lastly, a mini review article of this Research Topic touches the hot spot of research dealing with nuclear waste storage. Zhang Q. et al. proposed a method to prevent hydrogen embrittlement via reduce of hydrogen by the induced hydrogen consuming microorganisms. Nevertheless, more work has to be conducted to test the efficiency of this approach.

It is crucial from an ecological perspective to comprehend the diversity, structure, and assembly of microbial communities in ecosystems, as well as their interactions with one another. Environmental restoration by microbial communities requires knowledge of the resistance and adaptive strategies of microbial communities to inorganic and organic contaminants in ecosystems, their interaction, and the detoxification mechanism. The metal solubilizing and adsorbing properties of microbial communities can be sustainable and efficient in the extraction of metals from solid materials (ores and e-waste) in the metallurgical industry and environmental remediation (Amendola and Acharjee, 2022; Ayilara and Babalola, 2023; Pakostova et al., 2024). Therefore, studies related to the enhancement of metal solubilization and metal absorption capacity of microbial communities are highly needed. Efficient use of microbial communities for environmental management and the metallurgical industry requires in-depth knowledge of the interaction between environmental systems and microbial communities, the role of microbes in environmental management, and the interaction of microbes with the geochemical and mineralogical components of ecosystems.

## Author contributions

RZ: Writing – original draft, Writing – review & editing.
